# Pharmacokinetic–Pharmacodynamic Model for the Testosterone-Suppressive Effect of Leuprolide in Normal and Prostate Cancer Rats

**DOI:** 10.3390/molecules23040909

**Published:** 2018-04-15

**Authors:** Dong-Seok Lee, Sook-Jin Kim, Go-Wun Choi, Yong-Bok Lee, Hea-Young Cho

**Affiliations:** 1 College of Pharmacy, CHA University, 335 Pangyo-ro, Bundang-gu, Seongnam-si, Gyeonggi-do 13488, Korea; movingstone0620@gmail.com (D.-S.L.); supia925@gmail.com (S.-J.K.); gwchoi153@gmail.com (G.-W.C.); 2 College of Pharmacy, Chonnam National University, 77 Yongbong-ro, Buk-Gu, Gwangju 61186, Korea; leeyb@chonnam.ac.kr

**Keywords:** leuprolide, sustained release depot, pharmacokinetics, pharmacodynamics, pharmacokinetic–pharmacodynamic model

## Abstract

This study developed the pharmacokinetic (PK)–pharmacodynamic (PD) model of the testosterone-suppressive effect of leuprolide for evaluation of the sustained release (SR) depot and leuprolide solution in rats with or without prostate cancer. Six groups of rats were divided by the routes of administration (intravenous and subcutaneous injection) and kinds of formulation (vehicle, leuprolide solution, and SR depot). The PK profile after subcutaneous injection of leuprolide solution could be well-described by the one-compartment model. The absorption rate constant, the total body clearance, and the volume of distribution were estimated at 16.67 h^−1^, 514.46 mL/h, and 487.40 mL. Using PK parameters in the solution-administered group, the PK model for the SR depot was developed. The PK–PD model was constructed by describing the testosterone-suppressive effect of leuprolide using the feedback turnover model. The response of testosterone after administration of each formulation was well described using this PK–PD model for the estimation of PD parameters (*EC_50_*, *E_max_*, *h*) and systemic parameters (*k_in_*, *k_out_*, *k_f on_*, *k_f off_*). The developed PK–PD model containing an inhibitory feedback system could successfully describe the testosterone-suppressive effect of leuprolide in the type of formulation. The PK–PD model developed would be useful for evaluating the formulation of similar drugs whose effect is regulated through the feedback mechanism.

## 1. Introduction

Peptide and protein drugs have been increasingly important in the pharmaceutical industry, not only because of the advances in biotechnology and genetic engineering, but also because of better understanding of their role in physiology and pathology [1]. Peptide drugs are defined as molecules containing fewer than 50 amino acids and have many advantages [2]. They bind to their specific targets, resulting in high potencies of action and relatively few off-target side effects. Peptide drugs are fine-tuned to interact specifically with biological targets, evolving into potent endogenous hormones, growth factors, neurotransmitters, signaling molecules, and immunologic and defense agents [3]. Peptide drugs could have many valuable applications in clinical medicine, but so far applications of chemically synthesized peptides have been severely limited by their low systemic stability, high clearance, poor membrane permeability, and high costs of manufacturing [4].

Leuprolide (Pyr-His-Trp-Ser-Tyr-D-Leu-Leu-Arg-Pro-NHEt) is a hormone analogue composed of nine peptides synthesized for the treatment of sex hormone-related disorders [5,6]. It acts as a super-agonist of the pituitary gonadotropin-releasing hormone (GnRH) receptor in the hypothalamo–pituitary–gonadal axis and disrupts the maintenance of the normal hypothalamo–pituitary–gonadal axis and desensitizes the GnRH receptor [7]. This mechanism down-regulates the secretion of luteinizing hormone and follicle-stimulating hormone, resulting in lower levels of estradiol and testosterone in the blood [8]. Because of these characteristics, leuprolide is used to treat prostate cancer, endometriosis, fibroids and precocious puberty [9]. Prostate carcinoma is the development of cancer in the prostate, a gland in the male reproductive system [10]. Prostate cancer has the highest incidence among the reproductive cancers, accounting for 15% of the cancers that men have been diagnosed with [11]. In the United States, prostate cancer is the most frequently occurring cancer in men, and the number of deaths takes second place after lung cancer. Moreover, the incidence of prostate cancer has been steadily increasing because of a change of diet, such as high-fat diets and lack of fiber intake, and the increase in the aging population [12]. As mentioned above, leuprolide treats and delays prostate cancer, thereby maintaining a low concentration of plasma testosterone, but has trouble gaining optimal therapeutic effect, because of its low absorption rate, short duration of action by proteolytic enzyme, and high metabolic rate [8]. For these reasons, many kinds of formulations for sustained release of leuprolide were developed recently by the pharmaceutical industry, such as Lucrin depot^®^, Eligard^®^ and so on [7]. However, to achieve the optimal therapeutic effect of leuprolide, a more specific and specialized pharmacokinetic (PK)–pharmacodynamic (PD) model, including the characteristics of formulation and route of administration, is essential. Many models for some peptide drugs have been developed during the past several decades, but the PK and PD study of leuprolide has been lacking.

Several researchers have studied PK and PD evaluation after the administration of leuprolide [13]. Leitner et al. [7] investigated the PKs and PDs of several leuprolide sustained-release (SR) depots. But they evaluated only the PKs and PDs of leuprolide without developing a PK–PD model to identify the relationship between the PKs and PDs of leuprolide. Recently, interest in the PK–PD model has increased in terms of using the development process of the drug delivery system as a tool of evaluation of new formulations. In particular, the PK–PD model reflected the different release patterns of new formulations such as SR injectable depot is limited. Therefore, we aimed to develop a PK–PD model of the testosterone-suppressive effect of leuprolide in order to provide a comprehensive and quantitative description of leuprolide and testosterone, and to evaluate the PKs and PDs of leuprolide solution and leuprolide SR in normal and prostate cancer-induced rats using our developed model.

## 2. Results

### 2.1. In Vitro Release Evaluation of Sustained Release (SR) Depot

**Figure 1:**
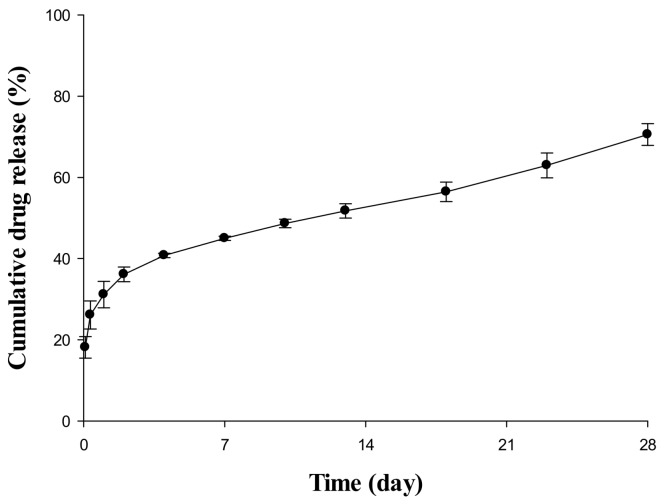
In vitro release profile of SR depot for 28 days in pH 6.8 phosphate-buffered saline (PBS) containing 0.02% Tween 80.

The result of an *in vitro* release test to confirm the release pattern of an SR depot in aqueous systems is depicted in Figure 1. The drug was already 10–20% released at the start of the release test, and a rapid release pattern was observed with a steep curve up to 4 or 5 days after the initial release. From 7 days to the last sampling time, it was constantly released at a very slow rate. Based on this dissolution pattern, this study divided the drug encapsulation pattern into a non-encapsulated section, a relatively fast-releasing section, and a slow-releasing section at a constant rate. In addition, the model was constructed by dividing each region, and the release rate and ratio of the drug were estimated through a model-fitting process because the *in vitro* and *in vivo* releases in all species were not directly correlated.

### 2.2. Pharmacokinetic Evaluation of Leuprolide

The PK parameters for each experimental group were estimated by one-compartmental analysis using WinNonlin^®^ software (version 7.0, Pharsight^®^, a Certara™ Company, Princeton, NJ, USA). Table 1 displays the estimated PK parameters in leuprolide solution-administered groups. Leuprolide showed 0.52 ± 0.10 h of the elimination half-life (t_1/2_), 1.42 ± 0.24 h^−1^ of *k* (elimination rate constant), and 105.50 ± 17.27 h·ng/mL of the area under the plasma concentration–time curve (AUC_0–∞_) after intravenous (IV) administration of leuprolide. In the case of subcutaneous (SC) injection, PK parameters were 0.66 ± 0.03 h of t_1/2_, 1.06 ± 0.55 h^−1^ of *k*, 16.67 ± 2.55 h^−1^ of *k_a_* (absorption rate constant), and 53.33 ± 9.96 h·ng/mL of AUC_0–∞_, respectively. The bioavailability of leuprolide after the SC administration was calculated to be 50.60%.

### 2.3. Pharmacodynamic Evaluation for the Testosterone-Suppressive Effect

In the vehicle-administered group with or without prostate cancer (Groups 2 and 5), the mean basal concentrations of testosterone in plasma were 4.35 ± 1.45 and 4.09 ± 0.97 ng/mL (± SE, *n* = 5). There were no significant differences in the average basal testosterone concentrations in plasma before and after the vehicle administration to Wistar and Copenhagen (Iar:COP) rats (*p* > 0.05), respectively. To confirm the influence of the vehicle solution, the basal testosterone concentrations in plasma before and after administration were compared and analyzed. The dispersion solution did not induce any significant changes for the entire experimental period.

**Figure 2:**
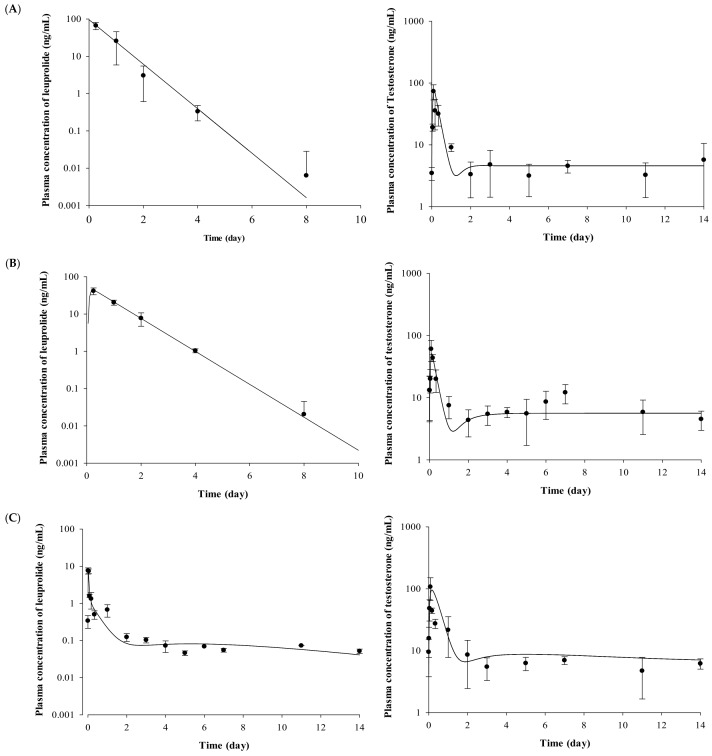

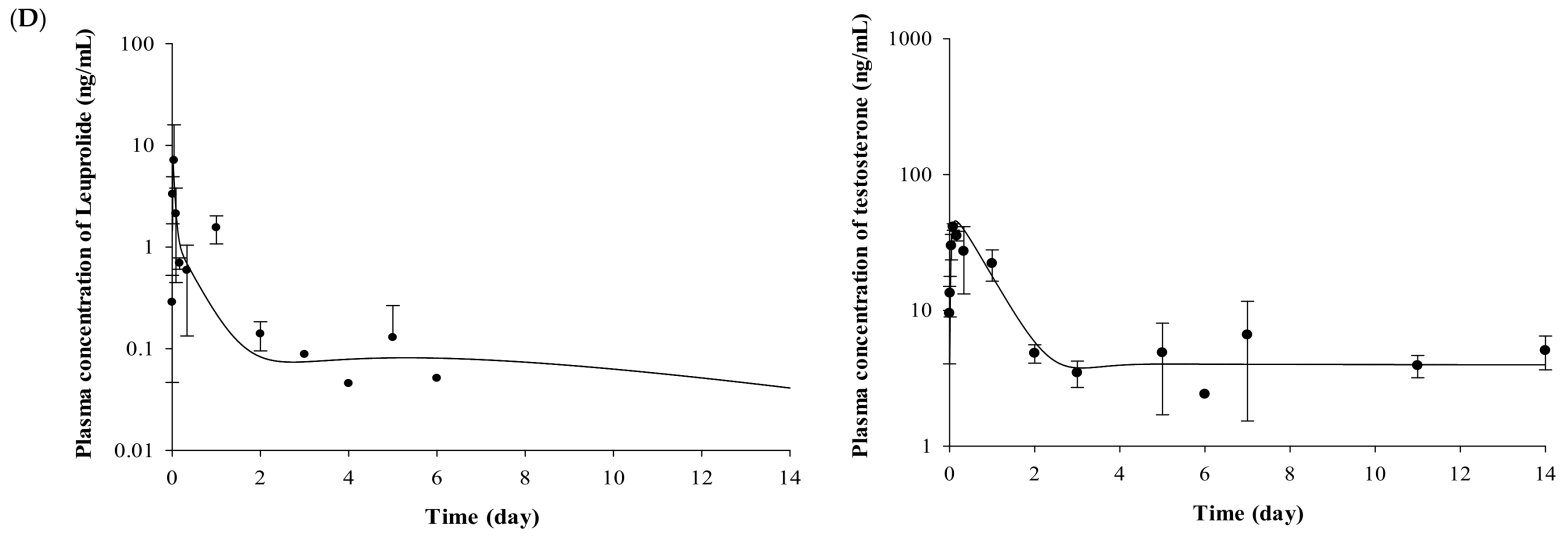
Mean observed concentration and fitted line of leuprolide and testosterone after (**A**) IV administration of leuprolide solution, (**B**) SC administration of leuprolide solution, (**C**) SR depot in normal Wistar rat, and (**D**) SR depot in Iar:COP rat using developed PK–PD model (*n =* 5). Closed circles indicate the observed leuprolide or testosterone levels in plasma. Solid lines mean the fitted line of leuprolide or testosterone by using developed PK–PD model.

The plasma concentrations of testosterone profiles after IV and SC administration of leuprolide solution and the Lucrin depot^®^ are shown in Figure 2. In the leuprolide solution and SR depot-administered group with or without prostate cancer (Groups 1, 3, 4, and 6), all the groups showed the “flare-up effect” [14], when the concentration of testosterone is dramatically increased. The maximal peak of testosterone level in each group, caused by the flare-up effect, was similar at 2 h after administration. After the peak of testosterone level in plasma, the concentrations of testosterone rapidly decreased in all the groups. However, there were different aspects of the testosterone levels after the time of minimal concentration of testosterone. Comparing the profiles of testosterone from 3 to 14 days among three groups (Groups 3, 4, and 6), the mean concentration of testosterone was 6.86 ng/mL for the leuprolide solution-administered group (Group 3), 5.93 ng/mL for the SR depot-administered group in the normal group (Group 4), and 4.39 ng/mL for the SR depot-administered group with prostate cancer (Group 6).

### 2.4. Pharmacokinetic–Pharmacodynamic (PK-PD) Modeling of Testosterone-Suppressive Effect of Leuprolide

The PK model for the solution-administered group was described by a one-compartment model. The equations describing the PK behavior of leuprolide are as follows:

(1) $$\frac{d\left(\mathrm{Drug}\right)}{dt}\;=\;\mathrm{dose}-k_{a}\cdot \mathrm{Drug}$$

(2) $$\frac{d\left(A_{p}\right)}{dt}\;=\;k_{a}\cdot \mathrm{Drug}-\frac{CL}{V_{d}}\cdot A_{p}$$

(3) $$C_{p}\;=\;\frac{A_{p}}{V_{d}}$$

The amount of leuprolide in plasma (*A_p_*) is absorbed from the drug compartment (Drug) and eliminated at a rate of *CL/V_d_*. The plasma concentration of leuprolide (*C_p_*) is calculated by dividing the *V_d_*.

The PK model for the SR depot was developed as divided into 3 sections: “non-capsuled release section” (NS), “diffusion-release section” (DS), and “erosive-release section” (ES). In the “non-capsuled section,” the behavior of leuprolide is assumed to be with the same as the behavior of the leuprolide solution. In this section, the PKs of leuprolide were described by the PK parameters estimated in the solution-administered group. In the second section, the “diffusion-release section,” the release of leuprolide depends on the drug entrapped in a polymeric matrix. In the “erosive release section”, leuprolide entrapped innermost releases with the slowest rate. To describe this slow release rate, the modified transit model was induced in the developed PK model. The equations for describing the PKs of the SR depot are as follows:

(4) $$\frac{d\left(NS\right)}{dt}\;=\;\mathrm{dose}\cdot N_{R}-k_{a}\cdot NS$$

(5) $$\frac{d\left(DS\right)}{dt}\;=\;\mathrm{dose}\cdot D_{R}-k_{d}\cdot DS$$

(6) $$\frac{d\left(ES_{1}\right)}{dt}\;=\;\mathrm{dose}\cdot E_{R}-k_{t}\cdot ES$$

(7) $$\left(N_{R}\;+\;D_{R}\;+\;E_{R}\;=\;1\right)$$

(8) $$\frac{d\left(ES_{2\cdots n}\right)}{dt}\;=\;k_{t}\cdot ES_{n-1}-k_{t}\cdot ES_{n}$$

(9) $$\frac{d\left(A_{p}\right)}{dt}\;=\;k_{a}\cdot NS\;+\;k_{d}\cdot DS\;+\;k_{t}\cdot ES_{n}-\frac{CL}{V_{d}}\cdot A_{p}$$

(10) $$C\;=\;\frac{A_{p}}{V_{d}}$$

in which *N_R_*, *D_R_*, and *E_R_* are the ratio of releasable leuprolide from each NS, DS, and ES in biological systems; *k_d_* is the diffusive release constant; and *k_t_* is the erosive release constant. The *A_p_* is absorbed from each section, NS, DS, and ES, at various release times (the lag time of drug release in DS, *t_lag,d_*; the lag time of drug release in ES, *t_lag_*_,*e*_). The PK parameters obtained from the developed PK model for SR depot are summarized in Table 2. All the PK parameters, including *N_R_*, *D_R_*, *E_R_*, *k_d_*, *k_e_*, *t_lag,d_*, and *t_lag,e_* of the two groups did not show any significant differences. The observed concentrations and fitted lines of leuprolide are depicted in Figure 2.

In Table 2, *ES_n_* means the number of transit compartments (ES). We have attempted to model 2 to 10 transit compartments (*ES_2_* – *ES_10_*) and obtained the best fit in 2 transit compartments; therefore, the number of transit models was fixed at 2. *N_R_*, *D_R_*, and *E_R_* were calculated differently in both species. Although formulations administered in Wistar and Iar:COP rats were the same lot of the same substance, they can be released differently depending on the physiological characteristics of species and individuals after the administration of drugs. Furthermore, in the case of sustained released drugs, physiological properties including the pH of the administered site and the pathophysiological state of the tissue could affect the swelling, degradation, dispersion, and wetting of the formulations. Also, the *k_d_* and *k_t_* used in this model are rate constants that include both the release process in the biological systems and the absorption process of the released drug, unlike the *k_a_* which contains only the absorption constant of the molecular state. In the diffusive section, dissolution is less influenced by physiological properties, since the dissolution process is relatively rapid. However, since the release in the erosive section proceeds very slowly, the formulations are greatly influenced by physiological properties such as pH during release over several weeks, and *k_t_* was estimated differently in Wistar and Iar:COP rats.

**Figure 3:**
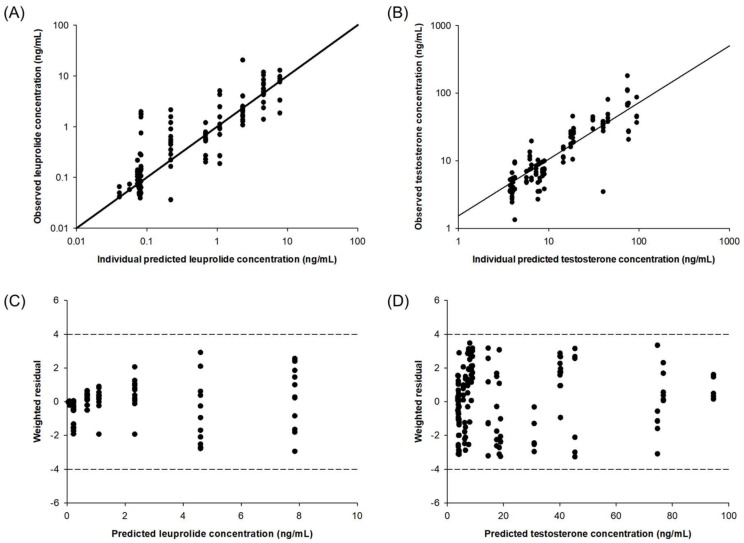
Diagnostic plots of the PK–PD model for leuprolide and testosterone: correlation of individual observed versus predicted concentration for leuprolide (**A**) and testosterone (**B**). Plots of weighted residual versus predicted concentration of leuprolide (**C**) and testosterone (**D**).

The diagnostic plots of the final PK–PD model are shown in Figure 3. The individual predicted versus observed concentration of leuprolide and testosterone obtained from the developed PK–PD showed good agreement. The distribution of the weighted residuals is narrow and close to the zero line on both sides. The value of weighted residual was within ± 4, and this means a suitable prediction model. The developed PK–PD model reasonably well described the observed data.

## 3. Discussion

In this study, the area under the effect curve (AUEC) was calculated for the quantitative analysis of PD evaluation for the testosterone-suppressive effect of leuprolide. Leuprolide is a GnRH agonist for the treatment of prostate cancer which is a target disease in this study. In the label of the Lucrin depot, this is indicated for palliative treatment of advanced prostatic cancer. Therefore, we used the testosterone concentration as a PD marker of prostate cancer, and plasma testosterone is one of qualified biomarkers used as outcomes in the therapeutic area of oncology [15]. Since most of the PD responses are expressed as a concentration profile of endogenous substances, it is difficult to calculate the value as a general trapezoidal rule. Scheff et al. [16] introduced several methods for calculating AUEC when baselines differ. In this study, drug-specific AUEC was calculated by subtracting the baseline AUEC from the AUEC calculated by a general method, and these drug-specific AUECs were calculated by dividing them into positive AUECs and negative AUECs. Positive AUEC is a parameter that corresponds to the “testosterone flare-up effect” of leuprolide when AUEC is greater than the baseline AUEC. Negative AUEC is a parameter corresponding to the testosterone-suppressive effect of leuprolide when AUEC is less than baseline AUEC. The positive and negative AUEC of each group is shown in Table 3. There were significant differences between positive and negative AUEC in all experimental groups. When absolute values of positive and negative AUEC were compared, negative AUEC was more than three times higher. This indicates that the testosterone-suppressive effect of leuprolide was predominately more effective than the flare-up effect of leuprolide. However, there was no significant difference in AUEC between each group on formulation, species and route of administration. This estimation of AUEC means that it could be used as a method for evaluating the formulation.

Leitner et al. [7] described the PKs of the SR depot by a four-step release curve containing the immediate release phase, diffusion-release phase, mixed diffusion–erosion phase, and pure erosive-release phase. The immediate release phase is the release aspect of the free drug, not trapped for the first 2 to 3 days. The diffusion-release phase is the comprehensive release that covers the continuous and delayed diffusion of the drug toward the surface of the microsphere after the initial peak. In the mixed diffusion–erosion phase, the diffusive release is decreased, and the erosion of the polymeric matrix is increased. Last, the release of the drug occurred through the erosion of the polymeric matrix in the pure erosive-release phase.

The PK–PD model, which describes the mechanism of negative feedback of leuprolide, was selected based on leuprolide mechanism characteristics and biomarker characteristics. First, the baseline model was developed in order to simulate the concentration of testosterone (*R*) when the drug did not affect the profile of testosterone. The equations of the baseline model are as follows:

(11) $$\frac{dR}{dt}\;=\;k_{in}-k_{out}\cdot R$$

(12) $$R_{0,\;wistar}\;=\;4.353$$

(13) $$R_{0,Iar:COP}\;=\;4.094$$

where *k_in_* and *k_out_* represent the apparent zero-order rate constant for the production of response and the first-order rate constant for the loss of response, respectively. These were estimated by curve fitting with the vehicle-administered group and used as the basic parameters of the drug-effect model described below (Group 2).

Subsequently, the drug-effect model was established based on *k_in_* and *k_out_* on the baseline model to identify the relationship between the PKs and PDs of leuprolide. For this, several PD models were attempted, and the final PD model decided on was the tolerance model including the feedback compartment. The equations of the developed PD model are as follows:

(14) $$\frac{dR}{dt}\;=\;\left(1\;+\;C_{E}\right)\cdot k_{in}\cdot F-k_{out}\cdot R$$

(15) $$\frac{dF}{dt}\;=\;\frac{k_{f,on}}{R}-k_{f,off}\cdot F$$

(16) $$C_{E}\;=\;\frac{E_{max}\cdot C^{h}}{C^{h}\;+\;EC_{50}^{h}}$$

where k*_f,on_* and *k_f,off_* represent the first-order rate constant for onset and offset of inhibition, *EC*_50_ is the leuprolide concentration producing half of the maximum effect, *E_max_* is the maximum effect capacity of the leuprolide, and *h* is the constant of the Hill equation for the interpretation of the relationship between the total concentration of leuprolide in plasma (*C*) and effect site (*C_E_*).

The developed PK–PD model well described the relationship between the leuprolide concentration and the testosterone-suppressive effect of leuprolide. Table 4 and Figure 2 present the parameters used for the developed model and simulation results. There were no significant differences in the estimated PK parameters (*p >* 0.05) between Wistar and Iar:COP rats, but there were significant differences in PD parameters, such as *E_max_* and *EC*_50_, between Wistar and Iar:COP rats (*p* < 0.05).

In this study, we tried several models to find the best model for the inhibition of testosterone by leuprolide. First, Bundgaard et al. [17] interpreted the feedback model of escitalopram using a dissociation constant in the PD model. We tried to simulate the testosterone inhibitory effect of leuprolide by introducing the dissociation constant in the study, but this attempt could not describe the decrease of the testosterone level below the basal level after the initial rise of testosterone. Romero et al. [18] described the hormone inhibitory effect of triptorelin using the Michaelis–Menten equation. When the negative feedback effect of leuprolide was simulated by introducing this equation into our model, the compensation effect of testosterone could be described. However, it could not describe the rapid increase and decrease of testosterone. The population PK–PD model of leuprolide in prostate cancer patients developed by Lim and Salem [19] is useful and applicable in clinical situations for personalized medicine. On the other hand, our model focused on the use of the PK–PD model as an evaluation tool during the development process of new formulations such as SR injectable depot formulation. In particular, in our study, the developed model reflects the release patterns of the leuprolide microsphere, so it can be used in variety of situations which are for comparative evaluation of the drug delivery system with different release patterns and for the prediction of the PK–PD relationship based on the release profile. Our model is simpler than Lim and Salem’s [20]. Continuing these trials and errors, when the constant of the Hill equation was introduced for describing the PD profile of testosterone, the compensation effect of testosterone could be expressed. Furthermore, the presence of the Hill constant was sufficient to express the rapid change of testosterone.

## 4. Materials and Methods

### 4.1. Chemicals and Reagents

**Figure 4:**
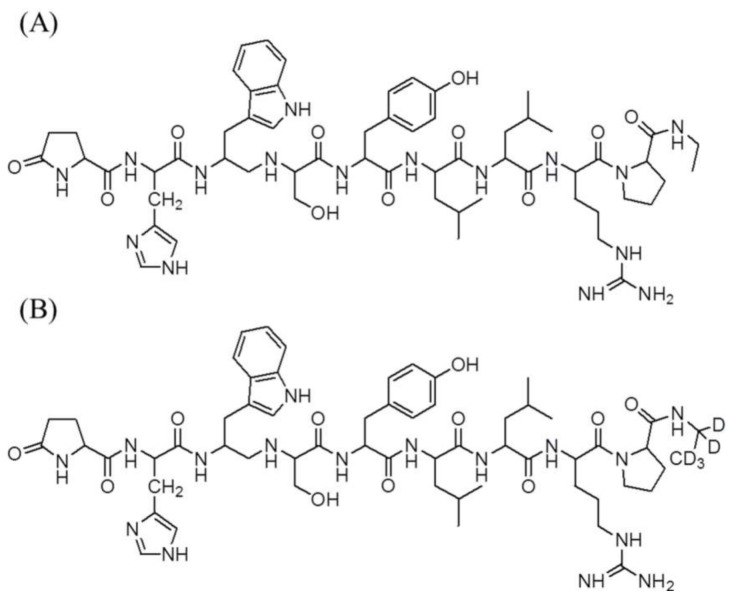
Chemical structures of (**A**) leuprolide and (**B**) leuprolide-d5 (IS).

The reference standards of leuprolide acetate (purity > 98%) and leuprolide-d5 acetate (purity > 97%) as the internal standard (IS) are shown in Figure 4. These were purchased from Toronto Research Chemicals (Toronto, ON, Canada). Lucrin depot^®^ (leuprolide acetate, 3.75 mg) was purchased from Abbvie Korea (Seoul, Korea). Acetic acid was supplied by Sigma-Aldrich (St. Louis, MO, USA). Acetonitrile, methanol, and methylene chloride were purchased from J.T. Baker (Phillipsburg, NJ, USA). High-performance liquid chromatography (HPLC)-grade water was obtained from an ElgaPurelab Option-Q system (ElgaLabWater, Marlow, UK) and used throughout this study. The other chemicals were of HPLC grade or better.

### 4.2. In Vitro Release Test of SR Depot

The release test for investigating release aspect of SR depot was conducted in a heating dry bath (confido–S20H, Seoul, Korea) at the speed of 300 rpm, and the temperature of 37 °C for 28 days. 10 mg of microsphere were added to 1 mL of phosphate-buffered saline (PBS) containing 0.02% Tween 80 as release media. At predetermined sampling times (2, 8 h, 1, 2, 4, 7, 10, 13, 18, 23, and 28 days), 0.8 mL of supernatant was collected after centrifugation at 3000× *g* for 3 min. Subsequently, 0.8 mL of fresh release media was supplemented to the polyethylene tube. The drug concentration in media supernatant was determined using high-performance liquid chromatography–ultraviolet (HPLC–UV).

### 4.3. Animals and Experimental Design

Adult male Wistar rats and Iar:COP rats with prostate cancer were obtained from Dae Han Biolink (Eumseong, Chungcheongbok-do, Korea) and Central Lab. Animal, Inc. (Seoul, Korea), respectively. Dunning observed the appearance of prostate cancer in male Iar:COP rats, and Dunning R-3327 adenocarcinoma is a spontaneously occurred prostate tumor found in male Iar:COP rats. In this study, Iar:COP rats were used for PK–PD studies in the prostate disease model [20]. All the rats were divided into six groups: a vehicle-administered group, a solution-administered group, and an SR-administered group, for rats with and without prostate cancer (5 rats per group) and were maintained on a 12 h dark–light cycle at ambient temperature (19 ± 1 °C) and relative humidity (50 ± 5%) with free access to water and food. This study was conducted according to the Guidelines for Ethical Conduct in the Care and Use of Animals and the rules of Good Laboratory Practice and was approved by the Institutional Animal Care and Use Committee (IACUC, No. 150035) at CHA laboratory animal research center. The rats were fasted for approximately 12 h with free access to water.

Either dispersion vehicle (composed of 0.5% carboxymethylcellulose sodium, 5% mannitol, and 0.1% Tween 80), 0.1 mg/kg of leuprolide acetate solution, and Lucrin depot^®^ equivalent to 0.1 mg/kg of leuprolide acetate was administered intravenously or subcutaneously to each experimental group according to an in vivo experimental design. For the vehicle-administered groups (Groups 2 and 5), the above dispersion solution was administered instead of the drug (Table 5). Blood samples were directly drawn from the right jugular vein at predetermined time intervals before administration and at 0.25, 1, 2, 4, 8 h, 1, 2, 3, 4, 5, 6, 7, 11, and 14 days after the drug administration. Approximately 300 μL of blood was collected periodically in heparinized tubes via the catheter and was immediately centrifuged at 12,000 *g* for 5 min and then stored at −80 °C until analysis.

### 4.4. Measurement of Leuprolide in Plasma Samples

The quantification of leuprolide in the plasma samples of Groups 1, 3, 4, and 6 was analyzed using a validated ultra-performance liquid chromatography–tandem mass spectrometry (UPLC–MS/MS) method, as described previously [21]. In total, 10 µL of IS solution (leuprolide-d5 10 ng/mL in 50% methanol), water (50 µL), and acetonitrile (1 mL) were added to 100 µL plasma in a microtube. After being shaken with a vortex mixer for 3 min and centrifuged at 10,000× *g* for 5 min at room temperature, the upper supernatant was transferred to a clean tube and mixed with 20 µL of acetic acid and 900 µL of methylene chloride by vortex-mixing for 3 min and centrifugation at 10,000× *g* for 5 min at room temperature. The aqueous upper sample aliquots (10 µL) were directly injected into the UPLC–MS/MS system. Analyses were performed with the following UPLC–MS/MS system: Acquity™ UPLC system (Waters Corp., Milford, MA, USA), Mass Spectrometer (Xevo™ TQ-S, Waters Corp.), HALO peptide ES-C_18_ column (2.1 mm × 100 mm, 2.7 µm particle size, Advanced Material Technology, Wilmington, DE, USA). The mobile phase consisted of 1% acetic acid in water and 1% acetic acid in acetonitrile with gradient at a flow rate of 0.2 mL/min.

The mass spectrometric detection was performed using an electrospray ionization (ESI) source in positive ionization mode. The optimization of MS parameters was accomplished as follows: capillary voltage, 2.9 kV; ion source temperature, 150 °C; desolvation temperature, 350 °C; flow rate of cone gas, 150 L/h; flow rate of desolation gas, 600 L/h; and pressure of argon gas, 4.5 × 10^−3^ mbar, respectively. The optimal collision energy was 27 eV for leuprolide and the IS. The multiple reaction monitoring transitions were accomplished as leuprolide (*m*/*z* 605.4→249.1) and IS (*m*/*z* 607.9→249.1). Data acquisition and analysis were performed by Masslynx 4.1 software (Waters Corp., Milford, CT, USA).

### 4.5. Measurement of Testosterone in Plasma Samples

The plasma concentration of testosterone in plasma samples of each group was quantified using testosterone enzyme-linked immunosorbent assay (ELISA) kits (R&D Systems, Minneapolis, MN, USA). The surface of each well was coated with goat anti-mouse antibodies, and horseradish peroxidase-labeled testosterone and unlabeled testosterone in plasma samples. Horseradish peroxidase-labeled testosterone reagent and unlabeled testosterone in the plasma samples would compete for binding these antibodies; for this mechanism, the intensity of color is in inverse proportion to the concentration of testosterone in the plasma sample.

Determination of the testosterone concentration was performed on using the validated Parameter^TM^ Testosterone Assay (R&D Systems, Minneapolis, MN, USA) and its process. First, 50 μL of primary antibody solution was added to each well, and then the microplate was incubated for 1 h at room temperature on a horizontal orbital microplate shaker (0.12″ orbit) set at 500 rpm. After the unbound reagents were washed away, the 100 μL of standard solution and plasma samples were added to each well, and 50 μL of testosterone conjugate reagent were subsequently added; the microplate was then incubated for 3 h at room temperature on a horizontal orbital microplate shaker (0.12″ orbit) set at 500 rpm. After being washed, 200 μL of substrate solution was added and the plate incubated for 30 min on the bench-top while protected from light. The reaction was stopped when 50 μL of stop solution (2N sulfuric acid) was added, and the optical density of each well was measured using SynergyMX (BioTek, Winooski, VT, USA). All plasma samples were measured twice. The standard curve was fitted using four-parameter logistic curve-fit and linear over the concentration range, 0.041–10 ng/mL for testosterone with the correlation coefficients ≥ 0.999. The detected sample concentrations above the 10 ng/mL were diluted and reanalyzed.

### 4.6. Estimation of PK, PD Parameters and Data Analysis

The PK and PD analysis was performed by non-compartmental and compartment analysis using WinNonlin^®^ software (version 7.0, Pharsight^®^, a Certara™ Company, Princeton, NJ, USA). The maximum plasma concentration (C_max_) and the time to reach C_max_ (T_max_) were measured by visual observation of the individual plasma concentration–time curve. The AUC_0–∞_ was integrated by the linear trapezoidal rule from time zero to the final measured concentration and was extrapolated from the final measured concentration to infinity. The clearance (CL) was calculated as the dose of leuprolide divided by AUC_0–∞_, and the t_1/2_ was calculated as 0.693/k. The elimination rate constant *k* is a value used to describe the rate at which the drug is removed from the biological system. The volume of distribution (V_d_), which is the theoretical volume in biological fluid, was calculated as CL divided by the *k*, and the bioavailability (F) was estimated as the AUC_0–∞_ obtained from SC administration divided by the AUC_0–∞_, obtained from IV administration. AUEC could be estimated by numerical integration over all segments of the effect-time curve by the trapezoidal rule without extrapolation to infinity. Because the endogenous testosterone was used as a PD marker in this study, the AUEC value subtracted from the baseline AUEC was used for data analysis.

All data calculated and estimated were analyzed for statistical significance by the Mann–Whitney U test with *p* < 0.05 indicating a significant difference.

### 4.7. Development and Evaluation of PK–PD Model of Leuprolide

**Figure 5:**
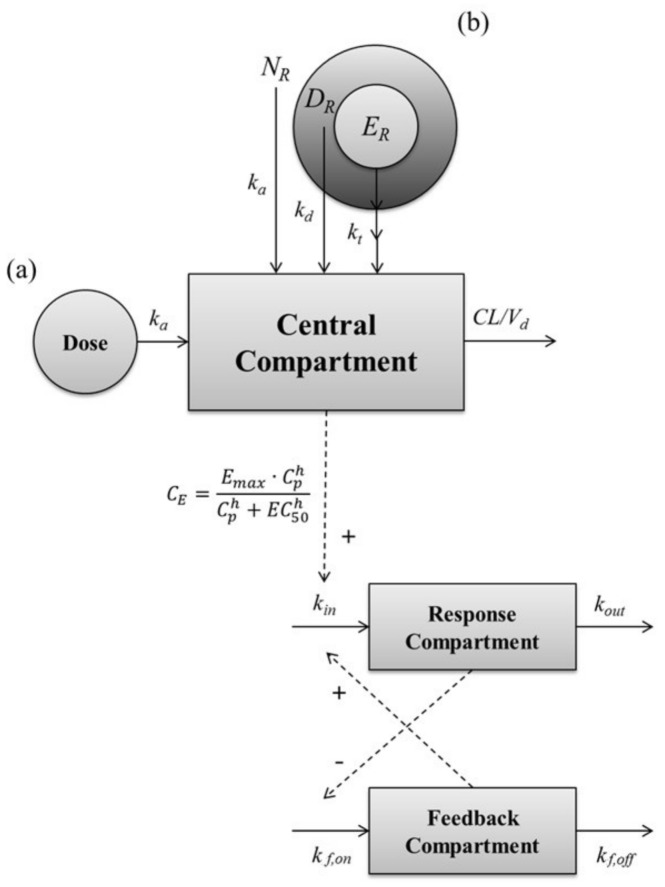
A schematic representation of the entire PK–PD model of leuprolide on the plasma testosterone level after SC administration of (a) leuprolide solution and (b) SR depot. Solid lines with arrows indicate the elimination or distribution of PKs, or indicate the production or elimination of responses. Dashed lines with arrows indicate the stimulatory or inhibitory effect of PDs. *N_R_*, the ratio of entrapped leuprolide in the non-capsuled section; *D_R_*, the ratio of entrapped leuprolide in the diffusive section; *E_R_*, ratio of entrapped leuprolide in the erosive section; *k_a_*, the absorption rate constant; *k_d_*, the diffusive-release constant; *k_t_*, the erosive-release constant; *CL*, the total clearance; *C_p_*, the plasma concentration of leuprolide; *C_E_*, the concentration of leuprolide in the effect site; *E_max_*, the maximum effective capacity; *EC_50_*, the half maximal effective concentration; *h*, the Hill equation constant; *k_in_*, the zero-order constant for production of response; *k_o_*_ut_, the first-order rate constant for loss of response; *k_f,on_*, the first-order constant for onset of inhibitory; *k_f,off_*, the first-order constant for onset of inhibitory.

The PK–PD modeling was conducted to describe the PK–PD relationship, and to estimate model parameters using Berkeley Madonna software (ver. 8.3.14). Berkeley Madonna software is a useful tool for modeling and analysis of various biological systems by solving the various differential equations. The PK analysis of leuprolide in the solution-administered group after IV and SC administration was carried out by non-compartmental and compartmental analysis, respectively. Based on the PK parameters such as *k_a_*, CL, and V_d_ in solution-administered groups, the PK model of the subcutaneous SR depot of leuprolide was developed. To describe the PK profile of the SR depot of leuprolide, we assumed that the SR depot consists of several sections containing the drug, and that these sections have different patterns of release. Consequently, the PD model was established in two steps. First, the baseline model was developed by using the baseline profiles of the vehicle-administered control group. Second, the drug-effect model was established based on the parameters that were estimated from the baseline model. Then the PD model was linked to the PK model to describe the effect versus time profile of leuprolide in rats with or without prostate cancer. To identify the relationship between the PK profile and the testosterone-suppressive effect of leuprolide, several PD models were tested, such as direct effect, indirect effect, turnover, tolerance, and rebound model. The structure of the developed PK–PD model for leuprolide is shown in Figure 5, and the linked PK–PD model was fitted by the classical Runge–Kutta method for interpreting the differential equations using Berkeley Madonna software.

Evaluation of the developed model was carried out based on graphical analysis using basic goodness-of-fit diagnostic plots. The adequacy of fitting was estimated to compare the individual observed and predicted values obtained from the final model. Random distribution of conditional weighted residuals was examined based on the differences between predicted and observed values.

## 5. Conclusions

This study was first carried out to develop the PK–PD model of the testosterone-suppressive effect of leuprolide on a rat model with or without prostate cancer. The PK and PD evaluation of each formulation, including the SR depot and the leuprolide solution, was conducted using the developed PK–PD model. The model, which contains the turnover feedback model, describes this response, and the parameters, including *k_in_*, *k_out_*, *h*, *k_f,on_*, *k_f,off_*, *E_max_*, and *EC_50_*, were estimated successfully. To conclude, the PK–PD model developed could help evaluate the formulation of drugs that have a feedback mechanism like that of leuprolide.

## Figures and Tables

**Table 1:** PK parameters of leuprolide after IV and SC administration of leuprolide solution (mean ± standard error (SE), *n* = 5).

Parameters	IV (Group 1)	SC (Group 3)
*k* (h^−1^)	1.42 ± 0.24	1.06 ± 0.55
*k_a_*(h^−1^)	-	16.67 ± 2.55
*CL* (mL/h)	248.61 ± 35.07	514.46 ± 40.10
*V_d_*(mL)	192.95 ± 54.24	487.40 ± 29.02
*C_max_*(ng/mL)	97.10 ± 13.04 ^a^	41.53 ± 4.61
t_1/2_ (h)	0.52 ± 0.10	0.66 ± 0.03
AUC_0-t_ (h·ng/mL)	103.44 ± 16.93	51.20 ± 9.56
AUC_0–∞_ (h·ng/mL)	105.50 ± 17.27	53.33 ± 9.96
F (%)	100	50.6

^a^ The concentration is the C_0_ (ng/mL) after IV administration.

**Table 2:** The estimated parameters used to predict the PKs of leuprolide in the SR-administered group using developed PK model (mean ± SE, *n* = 5).

Parameters	Wistar Rats (Group 4)	Iar:COP Rats (Group 6)
*ES_n_*	2	2
*N_R_*	0.18 ± 0.04	0.08 ± 0.02
*D_R_*	0.28 ± 0.07	0.43 ± 0.08
*E_R_*	0.54 ± 0.08	0.49 ± 0.08
*k_d_* (h^−1^)	0.08 ± 0.01	0.08 ± 0.01
*k_t_*(h^−1^)	0.0078 ± 0.0002	0.0193 ± 0.0083
*t_lag,d_* (h)	0.47 ± 0.09	0.35 ± 0.06
*t_lag_*_,e_ (h)	3.61 ± 1.53	2.58 ± 0.22

**Table 3:** The drug-specific AUEC separated by positive and negative AUEC for each group (mean ± SE, *n* = 5).

Group	Formulation	Routes	Animals	Positive AUEC	Negative AUEC
1	Leuprolide solution	IV	Wistar	208.24 ± 52.30	−1404.30 ± 287.31 *
3		SC	Wistar	220.87 ± 39.56	−775.98 ± 289.13 *
4	SR depot	SC	Wistar	407.91 ± 111.95	−761.48 ± 292.02 *
6		SC	Iar:COP	354.12 ± 78.95	−880.28 ± 298.31 *

* *p* < 0.05 between positive and negative AUEC after administration of leuprolide.

**Table 4:** The estimated parameters used to predict the pharmacodynamics of leuprolide using the developed PK–PD model (mean ± SE, *n* = 5).

Parameters	Wistar Rats			Iar:COP Rats
	Solution-IV (Group 1)	Solution-SC (Group 3)	SR-SC (Group 4)	SR-SC (Group 6)
*k_in_*(h^−1^)	0.68	0.68	0.68	0.35
*k_out_* (h^−1^)	0.16	0.16	0.16	0.06
*h*	2.00 ± 0.61	2.02 ± 0.05	2.00 ± 0.01	3.18 ± 1.27
*E_max_*	303.77 ± 12.90	183.50 ± 12.87	380.00 ± 87.54	634.50 ± 144.73 *
*EC*_50_ (ng/mL)	3.48 ± 1.74	6.17 ± 2.49	1.80 ± 0.57	3.34 ± 0.56 *
*k_f,on_* (h^−1^)	0.29 ± 0.19	0.14 ± 0.03	0.40 ± 0.25	0.083 ± 0.14
*k_f,off_* (h^−1^)	0.059 ± 0.017	0.02 ± 0.014	0.04 ± 0.032	0.45 ± 0.04

* *p* < 0.05 between Wistar and Iar:COP rats administered SR–SC.

**Table 5:** In vivo experimental design for developing the PK–PD model of leuprolide (*n* = 5).

Group	Animal	Route	Formulation	Dose	Sampling time
1	Wistar	IV	Leuprolide solution	0.1 mg/kg	0.25, 1, 2, 4, 8 h, 1, 2, 3, 4, 5, 6, 7, 11, 14 days
2	Wistar	SC	Dispersion vehicle	0.2 mL	8 h, 1, 7, 14 days
3	Wistar	SC	Leuprolide solution	0.1 mg/kg	0.25, 1, 2, 4, 8 h, 1, 2, 3, 4, 5, 6, 7, 11, 14 days
4	Wistar	SC	Lucrin depot dispersion	0.1 mg/kg (as leuprolide)	0.25, 1, 2, 4, 8 h, 1, 2, 3, 4, 5, 6, 7, 11, 14 days
5	Iar:COP	SC	Dispersion vehicle	0.2 mL	8 h, 1, 7, 14 days
6	Iar:COP	SC	Lucrin depot dispersion	0.1 mg/kg (as leuprolide)	0.25, 1, 2, 4, 8 h, 1, 2, 3, 4, 5, 6, 7, 11, 14 days
